# Influence of Parathyroid Hormone-Loaded PLGA Nanoparticles in Porous Scaffolds for Bone Regeneration

**DOI:** 10.3390/ijms160920492

**Published:** 2015-08-28

**Authors:** Piergiorgio Gentile, Vijay Kumar Nandagiri, Ritesh Pabari, Jacqueline Daly, Chiara Tonda-Turo, Gianluca Ciardelli, Zebunnissa Ramtoola

**Affiliations:** 1 School of Clinical Dentistry, University of Sheffield, Sheffield S10 2TA, UK; E-Mail: p.gentile@sheffield.ac.uk; 2 Department of Mechanical and Aerospace Engineering, Politecnico di Torino, Turin 10129, Italy; E-Mails: vijaympharm@gmail.com (V.K.N.); chiara.tondaturo@polito.it (C.T.-T.); gianluca.ciardelli@polito.it (G.C.); 3 School of Pharmacy, Royal College of Surgeons in Ireland, Dublin 2, Ireland; E-Mail: riteshpabari@rcsi.ie; 4 Division of Biology, Department of Anatomy, Royal College of Surgeons in Ireland, Dublin 2, Ireland; E-Mail: jdaly@rcsi.ie

**Keywords:** bone tissue, nanoparticles, parathyroid hormone, poly(lactide-*co*-glycolide), scaffolds

## Abstract

Biodegradable poly(lactide-*co*-glycolide) (PLGA) nanoparticles, containing human parathyroid hormone (PTH (1–34)), prepared by a modified double emulsion-solvent diffusion-evaporation method, were incorporated in porous freeze-dried chitosan-gelatin (CH-G) scaffolds. The PTH-loaded nanoparticles (NPTH) were characterised in terms of morphology, size, protein loading, release kinetics and *in vitro* assessment of biological activity of released PTH and cytocompatibility studies against clonal human osteoblast (hFOB) cells. Structural integrity of incorporated and released PTH from nanoparticles was found to be intact by using Tris-tricine SDS-PAGE. *In vitro* PTH release kinetics from PLGA nanoparticles were characterised by a burst release followed by a slow release phase for 3–4 weeks. The released PTH was biologically active as evidenced by the stimulated release of cyclic AMP from hFOB cells as well as increased mineralisation studies. Both *in vitro* and cell studies demonstrated that the PTH bioactivity was maintained during the fabrication of PLGA nanoparticles and upon release. Finally, a content of 33.3% *w*/*w* NPTHs was incorporated in CH-G scaffolds, showing an intermittent release during the first 10 days and, followed by a controlled release over 28 days of observation time. The increased expression of Alkaline Phosphatase levels on hFOB cells further confirmed the activity of intermittently released PTH from scaffolds.

## 1. Introduction

Bone regeneration is a complex cascade of biological events controlled by numerous bioactive molecules that provide signals at local injury sites allowing progenitors and inflammatory cells to migrate and trigger healing processes. Conventional tissue engineering strategies utilise combination of cells, biodegradable scaffolds and systemic administration of bioactive molecules to promote natural processes of tissue regeneration and development [1]. However, systemic administration of biomolecules such as growth factors often produces unpredictable results, probably due to their short biological half-life, lack of long term stability, tissue specificity and potential dose dependent carcinogenicity [2,3,4]. In addition to this, a well-timed and localised delivery of biomolecules from the scaffolds is necessary to achieve desired biomimetic effect [5,6].

Many methods for controlled delivery from scaffolds have been developed for use in bone tissue engineering. One of the most common methods of creating controlled and localised release is to utilise the physical properties of the scaffolds. This can be achieved by simple mixing of the target biomolecules with the materials during scaffold fabrication [7]. In such systems, the properties of the scaffold, such as pore size or crosslinking density could regulate their release rate by diffusion. The rate of scaffold degradation can affect their release over a time period [8,9]. Results from scaffold obtained by mixing are often un-satisfactory, because of the sensitivity of cells to the concentration of biological molecules and the short half-lives of these molecules. Several researchers have made to encapsulate biomolecules in polymeric particulate systems, such as micro/nanoparticles, and incorporate them into scaffolds for localised and/or controlled delivery [10]. However, when micro/nanoparticles incorporated into prefabricated porous scaffold, they often tend to coalesce or migrate, which may not serve the purpose of controlled/spatial delivery of biomolecules [11]. In this study, poly(lactide-*co*-glycolide) (PLGA) nanoparticles were incorporated during the fabrication of porous scaffolds (using freeze-dried method) based on chitosan (CH) and gelatin (G) blend, crosslinked with genipin (GP), to fix the location of nanoparticles within the scaffolds. Such a system primarily acts as a local regulator to control doses and kinetics of released growth factor, thus increasing their potential retention time at therapeutic concentration levels [12,13]. Chitosan was selected, as it is a biodegradable, biocompatible and non-toxic naturally derived polysaccharide which exhibits physiological inertness, haemostatic, antimicrobial and gel-forming properties [14]. CH scaffolds have been produced with a hydrophilic surface and cell adhesive/differentiating characteristics [15,16]. Furthermore, the inherent osteoconductive nature of CH empowers its potential use for bone tissue engineering applications [17]. Gelatin is a protein derived from collagen, and it has been frequently applied to artificial skin, bone grafts, and scaffolds for tissue engineering [18]. Its wide use in the biomedical field is motivated by the presence of Arg-Gly-Asp (RGD)-like sequences that promote cell adhesion and migration [19]. When CH and G are blended together, the formed structure can affect the spatial distribution of the integrin ligands of gelatin and the polycationic groups of CH interacting with the anionic cell surface. These effects influence cell adhesion, cellular bioactivity and tissue remodelling process and ultimately the quality of the regenerated tissue [20]. Furthermore, the mechanical properties and water stability of CH-G blend can be increased by crosslinking with suitable not cytotoxic crosslinking agents. In this work, GP, an aglycone derived from geniposide which is extracted from the fruit of *Gardenia jasminoides* Ellis, was selected as a crosslinker for CH and G blend [21]. PLGA nanoparticles has got an edge over the others because of its biocompatibility and known efficiency to deliver a number of growth factors, proteins or drugs in a time dependent manner both *in vitro* and *in vivo* [13].

The first aim of this work was focused on the optimisation process in the preparation of PLGA nanoparticles loaded with parathyroid hormone (PTH), selecting the appropriate biomolecule content.

Second aim was to incorporate the PTH embedded nanoparticles (NPTH) within porous chitosan-gelatin scaffolds, in order to characterise them for the localised PTH release and determine their biological activities by seeding clonal human osteoblast (hFOB) cells on scaffolds. PTH had been widely investigated for treating osteoporosis by adopting various delivery strategies such as injections, oral dosing, pulmonary inhalation, and transdermal delivery. But, most of these studies were focused on systemic delivery of PTH, whereas relatively few studies have looked at local delivery of PTH (1–34) [22,23]. Systemic administration of PTH does not allow maintenance of therapeutic concentrations over time. For instance, pulmonary inhalation of 18–20 μg PTH (1–34) gave a plasma concentration of approximately 45 ng/mL, but the duration was less than 120 min [24]. Consequently, continuous inhalations were required for achieving and maintaining effective concentrations. Alternatively, various strategies have been investigated to modulate PTH concentrations PLGA microspheres incorporated PTH (1–34) that were developed for local delivery [23]. The resultant microspheres sustained delivery of PTH from microparticles for more than 11 weeks. In another approach, Codrons *et al.* described a system for pulsatile delivery of PTH (1–34) by using alginate films [22]. PTH (1–34)/alginate films were alternated with polyanhydride layers inside a polylactic acid container. Release profiles showed 4 sharp peaks, with PTH (1–34) concentrations of 40–65 ng/mL, over a period of 4 days.

Comparing alternating and sustained exposure to the same PTH (1–34) concentration, intermittent exposure stimulated higher osteoblastic activity [25]. This could be expected, because continuous exposure down-regulates expression of its receptor in target cells [26]. PTH at physiological concentration is a potent suppressor of osteoblast differentiation; it is thought that PTH prevents differentiation of preosteoblasts into osteoblasts [27]. Although this bone-forming activity is antagonized, in part, by a stimulation of bone resorption, the net effect of intermittent PTH treatment is an improvement in bone microarchitecture and increased strength [28]. As a result, intermittent treatment with the 1–34 fragment of PTH is a Food and Drug Administration (FDA)-approved treatment modality for postmenopausal osteoporosis [29]. Localized delivery of PTH may produce different pharmacological activities and was therefore investigated in this paper.

## 2. Results and Discussion

### 2.1. Results

#### 2.1.1. PTH-Loaded Nanoparticles (NPTH)

##### Morphological Analysis of NPTHs

**Figure 1:**
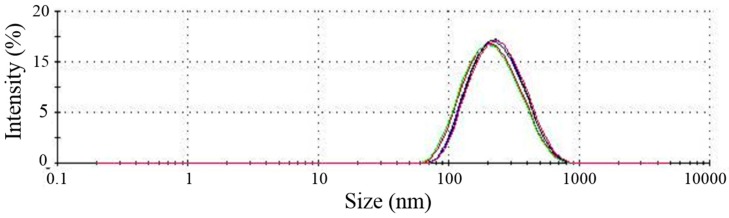
Particle size distribution for PTH-loaded nanoparticles (NPTH). The lines with different colors represents the repetitions of the tests.

**Figure 2:**
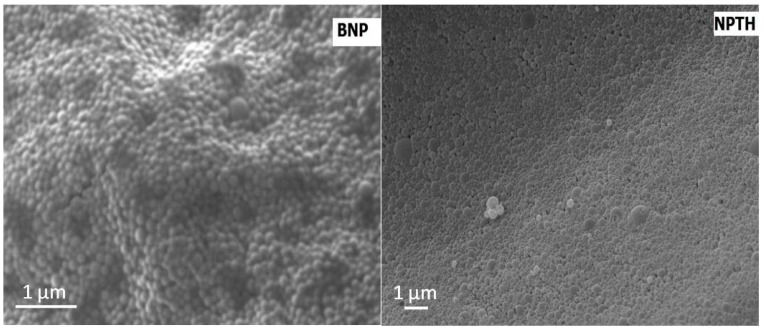
SEM image of bovine serum albumin (BSA) loaded poly(lactide-*co*-glycolide) (PLGA) nanoparticles (BNP, **left**) and NPTH_01 nanoparticles (NPTH, **right**).

Nanoparticles showed a median particle size of 205 nm and PDI of 0.23 (Figure 1). SEM images showed nanoparticles with regular spherical shape, smooth surface, and an absence of aggregation. Incorporation of PTH (1–34) did not affect the morphology of nanoparticles (Figure 2).

Nanoparticles prepared at PTH:BSA ratio of 1:10 showed a percentage of surface adsorbed and entrapped PTH of 77.9% and 22.1%, respectively (Table 1). As the ratio of PTH:polymer increased at 1:5 and 1:3.3, an increase in the surface adsorbed PTH (89.2% and 86.3% respectively) and a corresponding decrease in the entrapped PTH (10.8% and 13.7% respectively) was observed. Nanoparticles containing 2 mg of PTH in 100 mg of nanoparticles (NPs) (NPTH_02) showed highest encapsulation efficiency therefore was chosen for further studies.

**Table 1:** Amount of parathyroid hormone (PTH) detected in various formulations.

Sample	PTH Loading (%)	Encapsulation Efficiency (%)	Surface Adsorbed (%)	Entrapped (%)
NPTH_01 (1 mg PTH + 9 mg BSA)	2.6 ± 0.4	25.5 ± 1.5	77.9 ± 2.5	22.1 ± 2.5
NPTH_02 (2 mg PTH + 8 mg BSA)	6.7 ± 0.5	33.5 ± 0.8	89.2 ± 3.7	10.8 ± 3.7
NPTH_03 (3 mg PTH + 7 mg BSA)	7.1 ± 0.8	23.6 ± 0.9	86.3 ± 3.6	13.7 ± 3.6

##### Release Studies from Nanoparticles

**Figure 3:**
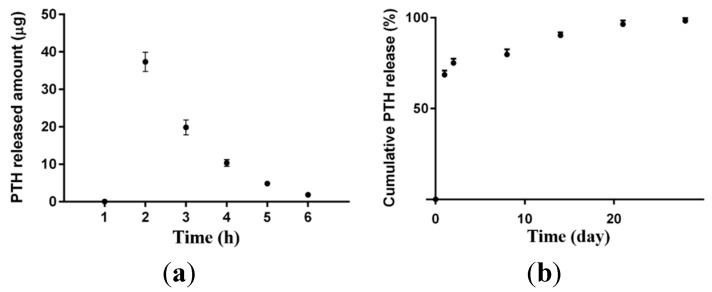
Cumulative PTH release from PLGA nanoparticles (**a**) over the initial 6 h; and (**b**) over 28 days.

PTH release from nanoparticles followed a biphasic pattern comprising an initial burst release of 75% during first 48 h, followed by a sustained release profile over 28 days. The initial burst release was related to the surface adsorbed PTH (Figure 3).

Proteins are generally considered to undergo denaturation when subjected to various stress conditions, such as sonication and homogenization [30].

**Figure 4:**
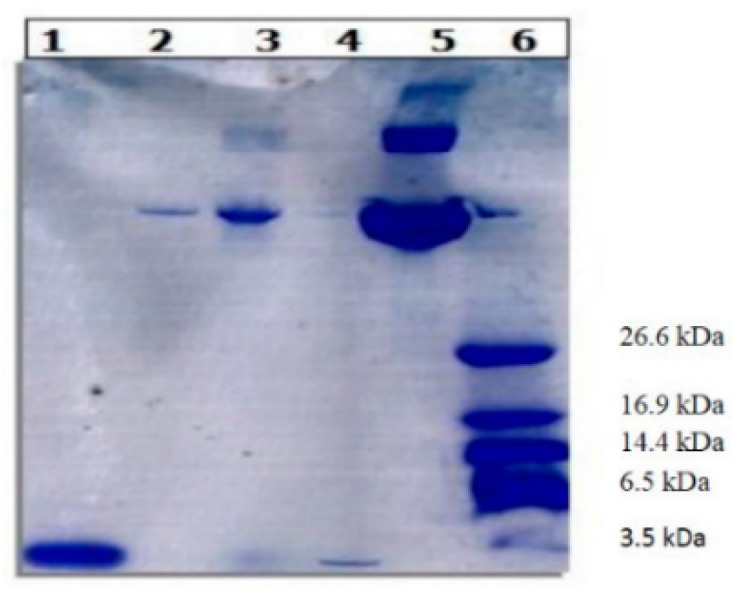
Tris-tricine SDS gel electrophoresis under reducing conditions.1, Pure PTH; 2, NPTH released; 3, entrapped NPTH; 4, Surface NPTH; 5, Pure BSA and 6, Molecular-weight size marker.

Therefore Tris-Tricine SDS PAGE was carried out under reducing conditions to evaluate structural integrity of PTH (Figure 4). The encapsulated PTH (both entrapped and on the surface) and released PTH from PLGA nanoparticles appeared to be intact as the standard PTH. However, the bands were appeared below 3.49 kDa band (of molecular weight marker) which could be attributed to the breakdown of PTH due to reducing conditions.

##### Cell Viability Test

**Figure 5:**
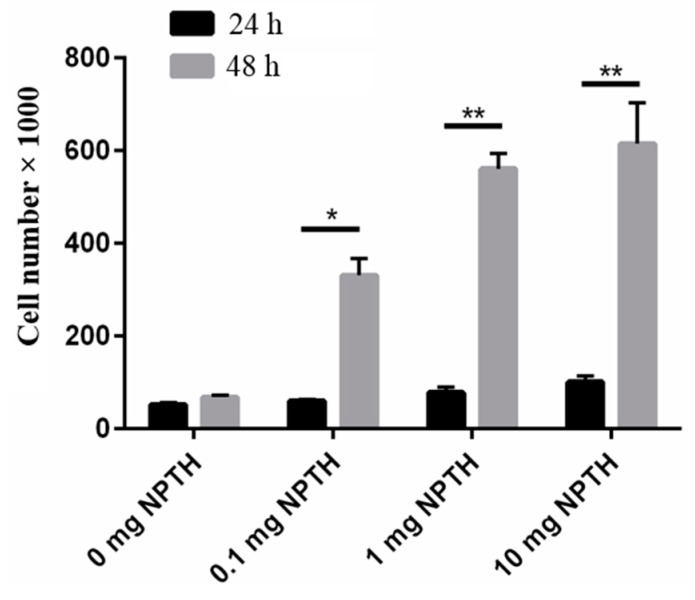
Effect of NPTH treatment on cell viability of hFOB cells. Bars represent the average cell number and error bars indicate standard deviation, *, *p* < 0.05 and **, *p* < 0.0001.

The effect of PTH (1–34) loaded PLGA nanoparticles on hFOB cell viability was evaluated and results show that PTH (1–34) loaded PLGA nanoparticles are cyto-compatable with hFOB cells (Figure 5). A dose dependent response was observed NPTH treatment initiated cell differentiation within 24 h of incubation and cell count significantly increased within 48 h of treatment, compared to untreated cells (0 mg NPTH).

##### *In Vitro* Bioactivity Assay for PTH (1–34) from Nanoparticles

**Figure 6:**
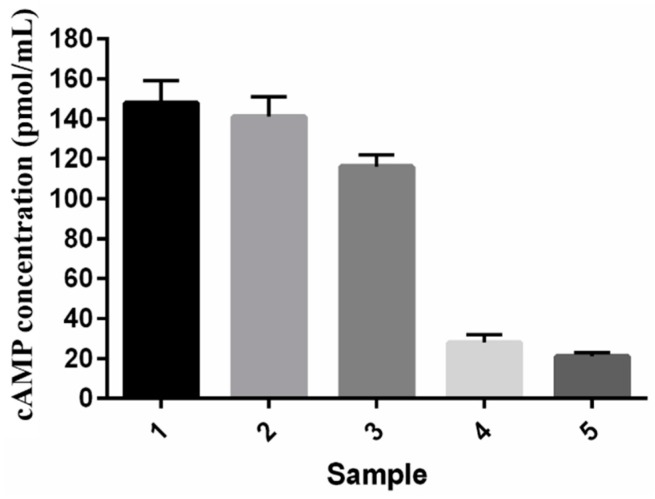
*In vitro* cyclic adenosine monophosphate (cAMP) activity on cell viability of hFOB cells: (1) Standard PTH (1–34); (2) PTH (1–34) extracted from NPTH; (3) NPTH released after 1 h; (4) NPTH released after 4 h and (5) released after 24 h.

*In vitro* PTH (1–34) bioactivity was measured by detecting the levels of 3,5 cyclic adenosine monophosphate (cAMP) in hFOB cell line by a cell-based competitive immune-assay. PTH (1–34) extracted from nanoparticles produced above 95% of bioactivity compared with the standard PTH (1–34), measured in terms of cAMP induction activity. PTH (1–34) released from NPTH displayed bioactivity of 75% after 1 h, 17% after 4 h release and 13% bioactivity after 8 h of release compared to the bioactivity of standard PTH (1–34) as shown in Figure 6.

**Figure 7:**
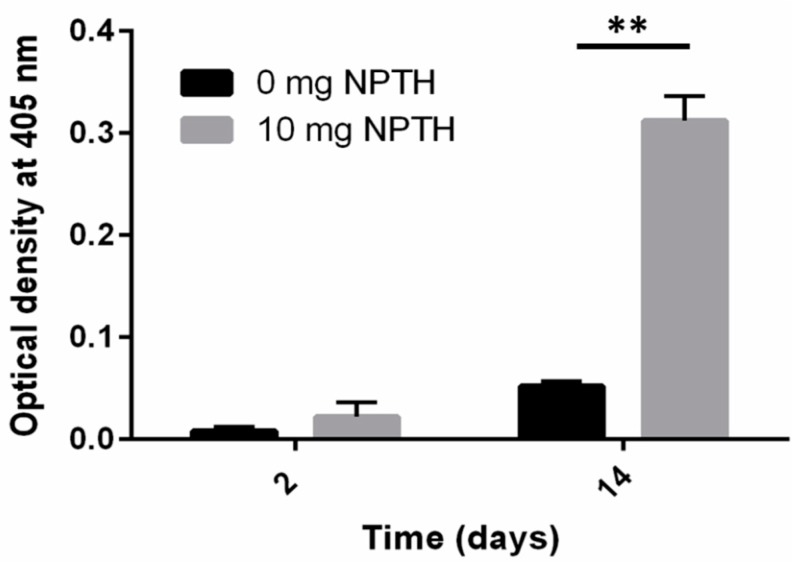
The ALP activity of hFOB cells in response to the treatment of NPTH. Bars represent the optical density at 405 nm and error bars indicate standard deviation, **, *p* < 0.0001.

The ability of NPTH to induce *in vitro* biomineralization in hFOB cells was evaluated by measuring the modified alkaline phosphatase (ALP) activity. The results reveal that NPTH induced *in vitro* biomineralization as the treatment time increased (Figure 7).

#### 2.1.2. CH-G Scaffolds Embedded with NPTHs

##### Scaffold Morphology

**Figure 8:**
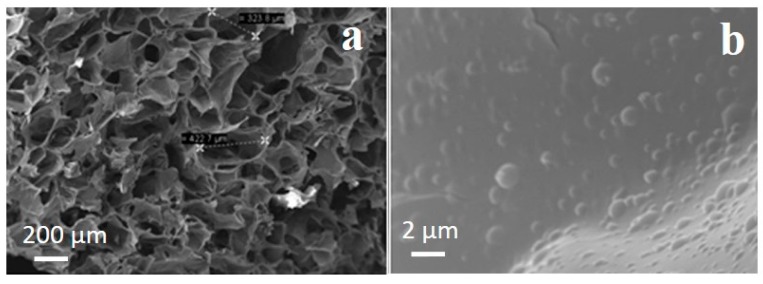
SEM images of (**a**) fractured sections of scaffolds embedding PLGA nanoparticles and (**b**) expanded section of (**a**) showing embedded nanoparticles. In the black boxes pores measurements are reported.

SEM micrographs of PLGA nanoparticles-embedded CH-G scaffolds (Figure 8) showed that PLGA nanoparticles were distributed on the pore walls, showing the formation of some aggregations of PLGA nanoparticles as the amount of particles increased (Figure 8b). Furthermore, the incorporation of nanoparticles into CH-G scaffolds did not change significantly the average pore size (146 ± 63 µm) loading as compared to the control (130 ± 37 µm, data not shown).

##### Dissolution Tests on NPTH-Loaded Scaffold

**Figure 9:**
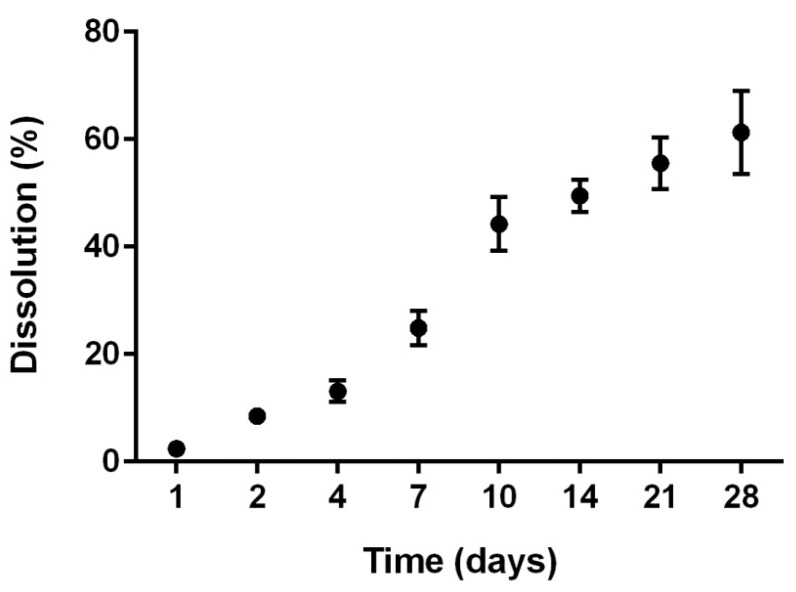
Dissolution behaviour of the NPTH-loaded porous scaffolds as a function of time. Measurements were carried out in PBS at pH 7.4 at 37 °C. Data are averaged on three measurements.

Dissolution tests were performed on NPTH-loaded scaffold, with the aim to study their stability in aqueous solution. CH-G scaffolds lost a weight of 2.4% ± 0.4% after one incubation day in Phosphate Buffered Saline (PBS) solution. Weight loss increased with time reaching a value of 61.3% ± 7.7% after 28 days showing a faster dissolution during the first 2 weeks (49.5% ± 3.0%) compared to the third and fourth week of incubation in PBS (Figure 9).

##### Release Studies from NPTH-Loaded Scaffolds

**Figure 10:**
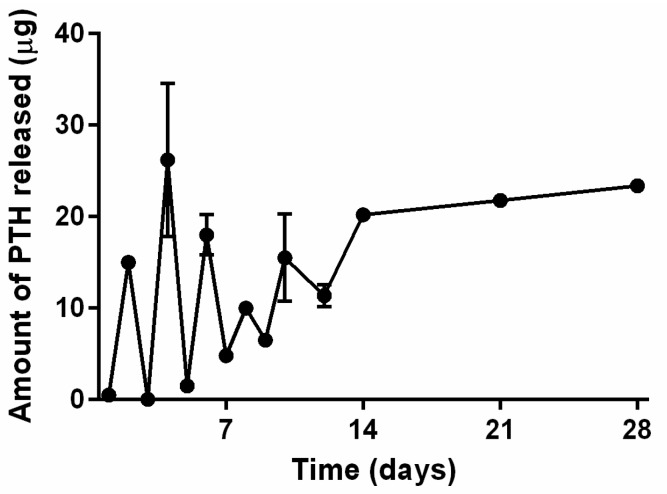
Release profile of PTH from NPTH engrafted scaffolds.

PTH release from NPTH engrafted CH-G scaffolds showed intermittent release up to day 10, where 0.5 μg (1.9%), 1.5 μg (5.8%), 10 μg (38.5%) of PTH was released at 24 h, day 5, day 8, respectively and 15.5 μg (59.6%) was released at day 10 (Figure 10). This was followed by controlled release where 80.8% and 100% of cumulative PTH was released at day 14 and day 28, respectively. May require to calculate cumulative release or else may need to smooth profile by trending line through data as opposed to join data points.

##### Cell Attachment and Viability Test

**Figure 11:**
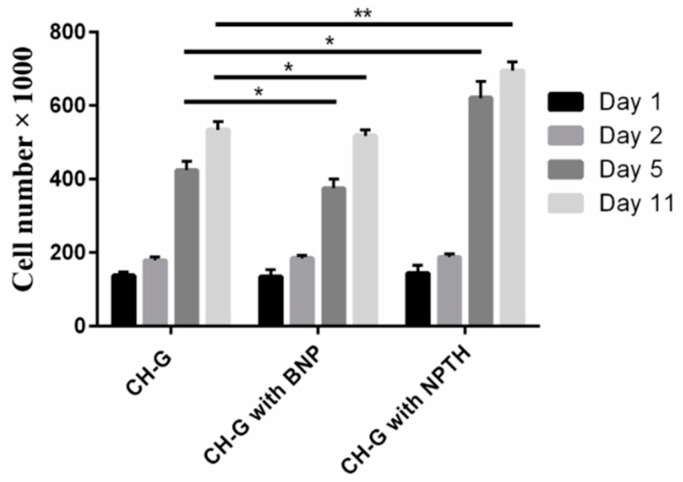
Metabolic cell viability of hFOB cells on scaffolds engrafted with various amounts of microparticles; day 1, 2, 5 and 11 post seeding of cells on scaffolds. Bars represent the average cell number and error bars indicate standard deviation, *, *p* < 0.05 and **, *p* < 0.0001.

Figure 11 showed no significant variation in cell viability in all scaffold groups for the first two days of culture, which indicates that the incorporation of PLGA nanoparticles does not affect cell attachment to the CH-G porous scaffolds. After 5 and 11 days, while scaffolds engrafted with PLGA nanoparticles showed slight reduction in cell number, the extent of cell growth was higher in scaffolds engrafted with NPTH.

##### *In Vitro* Biomineralization Test

**Figure 12:**
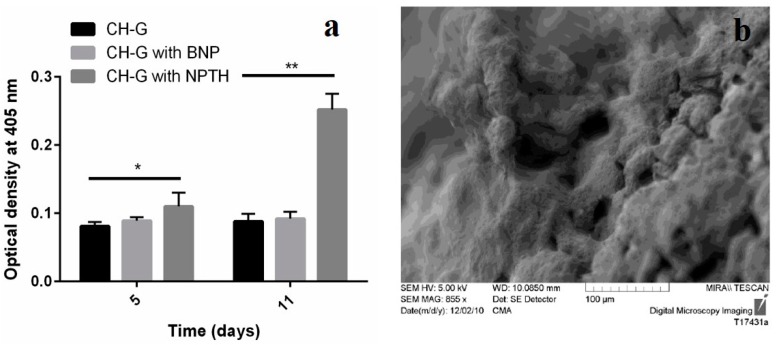
(**a**) ALP levels expressed on scaffolds; (**b**) SEM image representing bio-mineralization on NPTH engrafted scaffolds 11 days of post cell seeding. Statistical comparison by Kruskal-Wallis One Way Analysis of Variance on Ranks showed significant difference at *p* < 0.05 (*) and very significant different *p* < 0.0001 (**).

The results reveal that scaffolds engrafted with NPTH and induced modified alkaline phosphatase (ALP) levels post seeded with hFOB cells, which increased as the treatment time increased (Figure 12a). Furthermore, SEM image of NPTH engrafted scaffold confirms the Cauliflower like structure on surface which could be due to the calcification (Figure 12b).

### 2.2. Discussion

The recombinant form of the human PTH (1–34) has been reported to be a potent stimulator of bone growth. Intermittent doses of recombinant human PTH (1–34) applied systemically have an anabolic effect on cancellous bone, which results in increased bone mineral density [31,32,33]. One of the challenges in adopting PTH (1–34), for desired therapeutic effect, is maintaining its bioactivity during and after delivery. Intact PTH (1–84) and PTH (1–34) have plasma half-lives of less than 3 and 11 min, respectively. The rapid metabolic degradation of biologically active PTH (1–34) may make multiple administrations of PTH (1–34) necessary to maintain its effectiveness. This was also one of the main factors that has contributed to low bioavailability of PTH (1–34) by oral administration [22,34].

The 1–34 PTH-fragment with the preserved N-terminus still shows similar potency and pharmacological profile as the full length hormone PTH (1–84) [31]. In this study, PTH (1–34) was selected over the full length PTH (1–84) because PTH (1–34) is a clinically approved anabolic therapy for osteoporosis [35,36]. Furthermore, researchers have been demonstrated that localised delivery of PTH (1–34) enhances bone regeneration in rats. Therefore, local delivery of PTH (1–34) could be a means of achieving local bone healing at the site where it is needed, without the potential safety risks associated with systemic PTH (1–34) exposure [37,38]. PLGA microparticle based formulations have been investigated for the purpose of controlled/localized delivery of PTH (1–34) [39]. However, an optimal method of PTH (1–34) delivery has not been established. In this work, BSA/PTH loaded PLGA nanoparticles were prepared by double emulsion-solvent evaporation method, showing small particle size of 205 nm and narrow size distribution. Low entrapment of PTH in nanoparticles could be related to the diffusion of PTH during the secondary emulsion step and also during the solvent evaporation step, as PTH is a linear small peptide with a low molecular weight around 4 kDa protein. Higher amount of adsorbed PTH resulted in a high initial burst release within first 48 h of the study; followed by sustained release of entrapped PTH over 28 days. One of the most common causes for denaturation of protein during nanoparticles formulation could be various stressors such as sonication and homogenisation [30]. The activity of proteins depends on the tertiary and quaternary structure that allows the protein chains to fold and adopt a three-dimensional conformation [40]. SDS-PAGE carried out under reducing conditions indicates structural integrity of encapsulated and released PTH (1–34) from PLGA nanoparticles. Thus PTH (1–34) did not undergo structural damage during formulation and release from PLGA nanoparticles.

This could possibly be related to the stabilising effect of BSA attributed to their surface-active properties, which act against protein unfolding and aggregation during nanoparticle formulation steps. Albumins (*i.e.*, bovine, human or rat serum albumins) are reported to occupy the interfaces between organic phase and aqueous phase during emulsification step and shield the therapeutic protein from contact with the solvents or hydrophobic surfaces. Significant protein protection was reported when serum albumins were added to the inner aqueous phase during the primary emulsification step of multiple emulsion procedures [41]. In addition, BSA was thought to scavenge protons during polymer degradation, avoiding any aggregation resulting from acidity during the release of protein from PLGA microparticles [42,43]. It is evident from cell viability studies that PTH (1–34) loaded PLGA nanoparticles initiated cell differentiation within 24 h of incubation. The lowest number of cells were observed in control group (0 mg NPTH group) and at all NPTH amounts tested, there was an increase in cell number in a dose dependant manner, which could be attributed to delaying osteoblast apoptosis activity of released PTH (1–34) from all tested NPTH formulations. Furthermore, cell count were significantly increased within 48 h of treatment in all tested amounts of NPTH treated groups in compare to that of control cells (untreated cells) further demonstrates the anabolic effect of released PTH (1–34) from PLGA nanoparticles. The conformational state and structural integrity was further confirmed by assessing the biological activity of proteins. Since PTH (1–34) has been reported to induce an increase in cAMP level in osteoblastic cells [44], we examined the effect of equivalent amount of un-processed PTH (1–34) with that of total PTH (1–34) obtained from nanoparticles and released PTH (1–34) at different time intervals such as 1, 4 and 8 h on cAMP production in hFOB cells cultured in growth medium as a tool to detect the *in vitro* bio activity. These results indicate that PTH (1–34) loaded nanoparticles prepared by this method retained PTH (1–34) activity after the formulation and during the release. Thus confirms the structural integrity of encapsulated and released PTH. The bioactivity of encapsulated PTH (1–34) within PLGA nanoparticles was further confirmed by *in vitro* bio-mineralization study. ALP is a good marker for the differentiation of osteoblastic cells. The increased expression of ALP levels after NPTH treatment on hFOB cells further confirms the activity of encapsulated PTH (1–34).

Second part of this work reported the incorporation of 33.3% *w*/*w* of NPTH within porous CH–G scaffolds. From the SEM images, the nanoparticles were uniformly distributed throughout the walls of porous structures of the scaffolds. After a careful optimisation of the parameters into the scaffold preparation (*i.e.*, polymeric concentration (1%, 2%, 3% and 5% *w*/*v*), ratio between CH and G (1:1, 1:2, 1:3, 2:1, 3:1), genipin amount (1, 1.5, 2.5 *w*/*w*); data not shown), we selected the parameters described in the experimental section, in order to obtain a PTH intermittent release from the scaffolds, as it is evident from five discrete sharp peaks, with PTH (1–34) amounts of 0.5–26 µg, over a period of 10 days of release following which there was a controlled release over 28 days of observation time. This pulsatile release of PTH from scaffolds could be attributed to the distribution of particles within porous scaffolds and water holding capacity of scaffolds. NPTH particles which are associated at the surface of pores would have released PTH faster than that of the particles which were integrated through the walls of the porous structure of scaffolds. Furthermore, the dissolution studies revealed that around 45% of scaffolds weight loss after 10 days of incubation in PBS, due to probably the hydrolysis effect on the gelatin chain (less stable than the chitosan). Therefore, the scaffolds would have been disintegrated during 10 days of release study, leading to a leaching of particles from scaffolds. Hence, controlled release of PTH was observed after 10 days of release study.

Intermittent release of PTH within the scaffolds may stimulates proliferation and differentiation of osteoblasts [45]. To verify this concept, hFOB cells were seeded onto the porous CH–G scaffolds engrafted with NPTH. The results revealed a significant increase in cell number after five days of post seeding of scaffolds with cells. However, during first two days of cell viability there was no increase in cell number in case of scaffolds incorporated with particles rather there was a slight insignificant decrease, which could be attributed to the hydrophobicity of particle surfaces. Moreover, no significant amount of PTH was released during first day of release study (as witnessed in released study) in case of scaffolds engrafted with NPTH. But there was intermittent release of PTH during the first five days of release study and correspondingly, there was a significant cell growth and it was increased further during the 11 days observation period which could be attributed to the increased cell differentiation and proliferation as a consequence of non-continuous release of PTH. The increased expression of ALP levels after NPTH treatment on hFOB cells further confirmed the activity of intermittently released PTH from scaffolds.

## 3. Experimental Section

### 3.1. Materials

Recombinant human parathyroid hormone (PTH (1–34)) acetate (4117.8 Da) was supplied by Polypeptide Laboratories, San Diego, CA, USA. poly(lactic-*co*-glycolide) (PLGA; lactide:glicolide (50:50) 50:50, *M*_W_ = 48,300 Da) was obtained from Boehringer Ingelheim Pharma GmbH & Co. KG, (Ingelheim, Germany). Fraction V bovine serum albumin (BSA), ethyl acetate, polyvinyl alcohol (PVA), trehalose, acetonitrile CHROMASOLV^®^ (for HPLC grade) and sodium chloride (>99%) were all supplied by Sigma–Aldrich, Arklow, Ireland. Trifluoroacetic acid and all the remaining laboratory reagents were supplied by Fischer Scientific, Dublin, Ireland.

### 3.2. Preparation and Characterisation of PTH (Parathyroid Hormone) Loaded PLGA (Poly(lactide-co-glycolide) Nanoparticles (NPTH)

#### 3.2.1. Synthesis of NPTHs

PTH (1–34) loaded PLGA nanoparticles were prepared using a modified double emulsion-solvent diffusion method as previously described by Nandagiri [13]. Briefly, 1 mL of aqueous phase containing 10 mg of total protein (PTH and BSA were at ratios 1:9, 1:4 and 1:2.33 respectively), containing 3% (*w*/*v*) trehalose in PBS, was added to 4 mL of 25 mg/mL PLGA solution in ethyl acetate and probe sonicated for 2 min at 50 W (Branson sonifier 150, Branson Ultrasonics Corporation, Danbury, CT, USA). The resulting emulsion was transferred into 4 mL of 2.5% (*w*/*v*) PVA (pH 8.5) solution and sonicated further for 2 min to form secondary emulsion. This mixture was transferred into 25 mL of 1% (*w*/*v*) PVA (pH 8.5) solution and homogenized for 3 min at a speed of 13,500 rpm to stabilise the double emulsion and facilitate the solvent diffusion. The organic solvent was evaporated by stirring the double emulsion with 25 mL of normal saline at 30 °C for 3–4 h (until the solvent was evaporated). The nanoparticles were collected by ultra-centrifugation at 30,000 rpm for 30 min (Ultracentrifuge Sorvall RC 5C plus, Ramsey, MN, USA), washed three times with purified water and freeze-dried (Freezone 6, Labconco: −57 °C, 0.03 mbar, 24 h).

#### 3.2.2. Characterisation of NPTHs

##### NPTH Morphology

Nanoparticles were characterised for particle size using a Zetasizer (Nano ZS/ZEN 3600, Malvern Instruments, Worcestershire, UK) and surface morphology using a scanning electron microscope (SEM) (LEO 1450 VP, Leo Electron microscopy Ltd., Cambridge, UK).

##### PTH (1–34) Loading and Encapsulation Efficiency

Surface adsorbed and entrapped PTH (1–34) content of PLGA nanoparticles was determined as described by Proos [46] and Sivadas [47]. To analyse surface adsorbed PTH, NPTH particles (10 mg) were suspended in 1 mL of PBS and sonicated intermittently (6 times) for 2 min at 50 W (Branson Sonifier 150, Branson Ultrasonics Corporation, Danbury, CT, USA), and agitated vigorously for 1 h. The suspension was then centrifuged at 30,000 rpm for 15 min and the supernatant analysed for PTH (1–34). To analyse entrapped PTH (1–34), the pellet was suspended in 1 mL of ethyl acetate and vortexed for 15 min to dissolve the polymer and to precipitate encapsulated PTH (1–34). The sample was then centrifuged at 12,500 rpm for 15 min and the supernatant containing polymer was discarded. To the pellet, 0.5 mL of PBS was added and vortexed for 15 min and then centrifuged as described earlier. The supernatant was collected and same procedure was repeated for three times with the pellet and all supernatants of PBS containing PTH (1–34) were pooled (1.5 mL) and analyzed by HPLC (Agilent Series 1120 HPLC, Agilent Technologies, Wilmington, DE, USA). The HPLC was equipped with a Gemini C18 column (5 μm, 25 mm × 4.6 mm, Phenomenex, Cheshire, UK) and a UV detector—(220 nm, Libra, Biochrom Ltd., Cambridge, UK). Following conditions were used, mobile phase A: 0.1% (*v*/*v*) tri-fluoroacetic acid (TFA) in water, mobile phase B: 0.08% (*v*/*v*) TFA in acetonitrile; flow rate: 1.5mL/min and column temperature: 60 °C. Gradient mobile phase conditions were employed as follows: gradient B from 24%–32% gradient 0 to 32 min, followed by 32%–48% in 8 min. Percent PTH loading and encapsulation efficiency were determined by using Equations (1) and (2) Each sample was analyzed in triplicate and results are indicated as an average of three.

(1) PHT loading = (Amount of PHT/Amount of nanoparticles) × 100

(2) Encapsulation efficiency = (PHT loading/Theoretical PHT loading) × 100

##### *In Vitro* PTH (1–34) Release Studies

*In vitro* PTH (1–34) release from NPTH particles (20 mg) was determined in 1 mL of PBS (pH 7.4) containing 0.02% *w*/*v* sodium azide (Sigma, Arklow, Ireland) as a preservative and under gentle shaking at 100 rpm at 37 °C in triplicate. At appropriate time intervals, samples were centrifuged for 25 min at 30,000 rpm and the supernatant was replaced by fresh 1 mL PBS containing 0.02% *w*/*v* sodium azide. The supernatant was assayed for PTH (1–34) content by using PTH-EIA (Bachem, Saint Helens, UK) kit according to the manufacturer recommendations. Absorbance measurements read at 450 nm recorded on a spectrometer (Varioskan Flash, version 4.00.53, using SkanIt Software 2.4.3 RE for Varioskan Flash software, Thermo scientific, Waltham, MA, USA) were used to calculate the PTH concentrations by the log-logit curve.

##### Tris-Tricine Sodium Dodecyl Sulfate Polyacrylamide Gel Electrophoresis (SDS-PAGE)

Structural integrity of PTH (1–34) extracted from NPTH was assessed by SDS-PAGE under reducing conditions using precast Novex^®^ 10%–20% Tricine Gelon XCell SureLock™ according to the protocol from Invitrogen, Carlsbad, CA, USA [30]. Dithiothreitol (DTT; NuPAGE Sample Reducing Agent, Invitrogen/Life Technologies, Carlsbad, CA, USA) is used for cleaving the disulfide bonds and thus breaks the protein in smaller molecular weights. After electrophoretic separation, the gel was stained for 4 h with Coomassie brilliant blue R-250 (0.008% in 10% acetic acid) at room temperature and destained using a solution containing 40% (*v*/*v*) methanol, 50% (*v*/*v*) water and 10% (*v*/*v*) acetic acid.

##### Cell Viability Studies

hFOB (ATCC, Manassas, VA, USA) pre-osteoblastic cells were cultured under standard conditions (5% CO_2_, 37 °C). Cells were routinely grown to 80% confluence in T175 culture flasks (Sarstedt, Ireland) containing culture media; a 1:1 ratio of Hams F12 and Dulbecco’s modified Eagle’s medium (without phenol red), 10% foetal bovine serum, 1% penicillin/streptomycin 10 mg/mL (Sigma–Aldrich, Arklow, Ireland). Expanded hFOB cells of passage 5–15 were harvested with trypsin-EDTA treatment, centrifuged and re-suspended in the culture medium. Cells were seeded into two 48 well tissue culture plates (one for 24 h and another for 48 h) at a cell density of 50,000 cells per well (250 µL of 200,000 cells/mL stock). The plates were incubated at 37 °C in humidified 5% CO_2_ overnight to allow the cells to attach to the bottom of the wells. The medium from each well was replaced with 250 μL of fresh medium and to each wells 50 μL of nanoparticle suspension containing different NPTH amount. The plates were placed on an orbital shaker (Biosan, Riga, Latvia) in the incubator at 37 °C and 5% CO_2_, and the assay was carried out at the end of 24 and 48 h, using Alamar blue assay kit (Invitrogen, Biosciences, Dublin. Ireland). The samples were incubated in an orbital shaker at 37 °C, (shaking rate of 50 rpm) for 4 h after which, 100 µL of the media was removed, transferred into a 96 well microplate and the absorbance at 570 and 610 nm of the media was read using a UV-visible spectrophotometer. The percentage of reduced dye as a function of cell viability was calculated in accordance with manufacturer’s recommendations. Samples were analyzed in triplicate for each nanoparticle concentration type.

##### *In Vitro* Bioactivity Assay for PTH (1–34)

Stimulation of cyclic AMP (cAMP) synthesis was used to determine the *in vitro* bioactivity of PTH (1–34) loaded nanoparticles as described by Shoyele *et al.* [48]. In detail, MC3T3-E1 (subclone 4) cells were cultured in minimum essential medium alpha medium (GIBCO, Invitrogen/Life Technologies, Carlsbad, CA, USA) with 10% fetal bovine serum and 100 units/mL of penicillin and streptomycin. Cells were plated at 50,000 cells/well in 12-well plates and induced to differentiate with the addition of ascorbic acid (50 μg/mL; Sigma, Arklow, Ireland) and α-glycerophosphate (10 mM; Sigma, Arklow, Ireland). The cells were used for the bioactivity assay after 5–7 days, when maximal PTH-1 receptor expression is reported to be occur. The confluent cells in each well were treated for 5 min with 500 μL of 0.5 mM of phosphodiesterase inhibitor, isobutylmethylxanthine (IBMX) (Sigma, Arklow, Ireland) in Kreb’s Ringer Buffer (KRB). Following treatment with IBMX, the cells were washed with KRB and subsequently treated with equivalent amounts of standard PTH (1–34), with PTH (1–34) extracted from NPTH-02 and NPTH-02 eluent for designated times points (1, 4 and 24 h) and incubated for 20 min at 37 °C. After incubation, cells were lysed using a lysis buffer (Cell Biolabs, San Diego, CA, USA) and incubated at 4 °C for 20 min. The cells were then scraped off the surface using a cell scraper. The cell suspension was then transferred to a centrifuge tube and stored at −20 °C until analysed for cAMP. The frozen samples in the centrifuge tubes were thawed and centrifuged at 10,000 rpm for 10 min. The supernatants were used for cAMP estimation. cAMP was measured using an enzyme immunoassay (EIA) kit (Biomedical Technologies Inc., Stoughton, MA, USA) following the manufacturer’s instructions.

##### *In Vitro* Bio-Mineralization Test on PTH-Loaded Nanoparticles

*In vitro* bio-mineralization induced by various amounts of PTH (1–34) loaded nanoparticles on hFOB cells was assessed by modified alkaline phosphatase activity (ALP) assay. Briefly, hFOB cells were seeded (2 × 10^4^ cells/well) on medium containing different NPTH amount in 24-multiwell plates. Growth media were replenished once every 2–3 days. The ALP assay was carried out at the end of days 5 and 14, using p-nitrophenyl phosphate disodium salt (pNPP reagent) (Sigma, Arklow, Ireland). Briefly, the NPTH were filtered and washed three times with PBS and 5 mL of PBS containing 20% *v*/*v* pNPP added to the washed scaffolds. Samples were incubated for 15 min after which, 100 µL of the supernatant from each well was plated in triplicate into a 96-well plate and their absorbance of the samples was read at 405 nm using a spectrometer (Varioskan Flash, version 4.00.53, using SkanIt Software 2.4.3 RE for Varioskan Flash software, Thermo scientific, Waltham, MA, USA). Enzyme activity was expressed as a function of optical density (OD) at 405 nm. The mean values of absorbance and the standard deviations were obtained from three different experiments.

### 3.3. Incorporation of NPTH within CH-G Scaffolds

NPTH nanoparticles embedded CH–G scaffolds were obtained by dispersing an aqueous suspension of PLGA nanoparticles into CH–G blend solution (3% *w*/*v*) at a concentration of 10 mg per mL of CH–G blend solution (33.3% *w*/*w* NPTH loading with respect to CH–G weight). CH–G scaffold without nanoparticles was considered as control scaffolds. The ensuing preparative stages, such as crosslinking, lyophilizing, and neutralizing were performed following the protocols as described previously by us, with the addition of 1.5% *w*/*w* of genipin into the polymeric solution [13].

#### 3.3.1. Dissolution Tests

To study the effect of nanoparticles loading on *in vitro* dissolution of scaffolds, cylindrical scaffold samples of 8 mm diameter and approximately 4 mm thickness were incubated in 3 mL of PBS (pH 7.4) for 28 days at 37 °C. The dissolution degree was calculated in terms of percentage weight loss, $W_{\text{L}}$ %) using the formula:

(3) $$W_{\text{L}}\left(\text{\%}\right)=\frac{\left(W_{10}-W_{0}\right)}{W_{0}}\times 100$$

where, *W*_10_ is the dry weight of scaffolds after 10 days of incubation in PBS and *W*_0_ is the initial weight. The values were expressed as the mean ± SD (*n* = 3).

#### 3.3.2. PTH Release from Scaffolds

PTH release from NPTH engrafted CH–G scaffolds was determined by incubating cylindrical scaffold samples of 8 mm diameter and approximately 10 mm thickness in a 10 mL vial containing 3 mL of PBS (pH 7.4) for 28 days at 37 °C. At predetermined time intervals, supernatant from the samples containing released PTH was withdrawn and replenished by 3.0 mL of fresh PBS solution. Samples were analyzed for PTH content by using EIA kit. The average and standard deviations were presented.

#### 3.3.3. hFOB Cell Seeding, Attachment and Viability on CH-G Scaffold

hFOB (ATCC, Manassas, VA, USA) pre-osteoblastic cells were cultured and harvested and seeded on scaffolds as described previously. At fixed time intervals (1, 2, 5 and 11 days), metabolic viability of hFOB cells on the scaffolds (control scaffolds, scaffolds with blank nanoparticles (BNP) and NPTH grafted scaffolds) was determined by Alamar blue dye. Samples were analysed in triplicate for each scaffold type. After collecting samples for Alamar blue assay, all scaffolds were washed three times by immersing them in sterile PBS and then incubated in fresh 5 mL growth medium.

#### 3.3.4. *In Vitro* Bio-Mineralization Test

*In vitro* bio-mineralization induced by PTH (1–34) loaded nanoparticles on hFOB cells seeded on scaffolds was determined at two time points such as day 5 and day 11 by ALP assay as described previously. Briefly, hFOB cells were seeded on CH-G scaffolds engrafted with MSIM particles to a final seeding density of 4 × 106 cells, placed in sterile 6-well plates with 5 mL of the growth medium and incubated. Growth media were replenished once every 2–3 days. The ALP assay was carried out at the end of days 5 and 11 as described before. Furthermore, the *in vitro* bio-mineralization was further confirmed by SEM topography analysis after 11 days of cell seeding.

### 3.4. Statistical Analysis

Experiments were run in triplicate for each sample. All data were expressed as mean ± SD for *n* = 3. Statistical analysis was determined by using Analyse-it v2.22 software (add-in for Microsoft Excel; Analyse-it Software, Ltd., Leeds, UK). The statistical differences between groups were calculated using Kruskal–Wallis One Way Analysis of Variance on Ranks (ANOVA). Statistical significance was declared as significant (*) at *p* < 0.05, and very significant (**) at *p* < 0.0001.

## 4. Conclusions

In this study, we successfully incorporated PTH within biodegradable PLGA nanoparticles in order to be incorporated within porous 3D scaffolds for the purpose of spatio-temporal release of bio active molecules. It is clearly evident from *in vitro* bioactivity (in terms of inducing cAMP), tristricine SDS PAGE and *in vitro* bio-mineralisation studies that PTH (1–34) remains structurally intact and active after encapsulation within, and release from nanoparticles. This is an important initial step in tissue engineering strategies aimed at the use of PTH (1–34) as an anabolic agent to improve bone regeneration. Furthermore, NPTHs showed an intermittent release within the porous scaffolds during the first 10 days and, followed by a controlled release over 28 days of observation. Finally, the increased expression of Alkaline Phosphatase levels after NPTH treatment on hFOB cells further confirmed the activity of intermittently released PTH from scaffolds.
